# A Prospective Analysis of Skin and Fingertip Advanced Glycation End-Product Devices in Healthy Volunteers

**DOI:** 10.3390/jcm11164709

**Published:** 2022-08-12

**Authors:** Dominik Adl Amini, Manuel Moser, Erika Chiapparelli, Lisa Oezel, Jiaqi Zhu, Ichiro Okano, Jennifer Shue, Andrew A. Sama, Frank P. Cammisa, Federico P. Girardi, Alexander P. Hughes

**Affiliations:** 1 Spine Care Institute, Hospital for Special Surgery, 535 East 70th Street, New York, NY 10021, USA; 2 Department of Orthopedic Surgery and Traumatology, Charité University Hospital Berlin, Charitéplatz 1, 10117 Berlin, Germany; 3 Department of Spine Surgery, Lucerne Cantonal Hospital, Spitalstrasse 16, 6000 Lucerne, Switzerland; 4 Department of Orthopedic Surgery and Traumatology, University Hospital Duesseldorf, Moorenstr. 5, 40225 Duesseldorf, Germany

**Keywords:** AGE, advanced glycation end products, skin autofluorescence, fingertip fluorescence, osteoporosis, connective tissue

## Abstract

Background: Advanced glycation end products (AGEs) have been shown to accumulate in bone and are gaining interest in connective tissue research. Aims: To investigate the intrarater reliability, two-timepoint agreement and correlations within and between two commercially available skin autofluorescence (SAF) AGE devices. Methods: Healthy volunteers were enrolled in a prospective study at a single academic institution. Each participant underwent SAF analysis by two different, commercially available devices on two occasions, 14 days apart. Upon enrollment, a general survey about the participant’s lifestyle and health status was completed and followed up on for any changes at timepoint two. Results: In total, 40 participants (F:M ratio 5:3) with an average age of 39.0 ± 12.5 years were analyzed. For the AGE reader (skin) and AGE sensor (fingertip), both intrarater reliability and two-timepoint agreement were excellent with an interclass correlation coefficient (ICC) > 0.90 and a strong correlation within both machines. However, there was no correlation between both machines for either timepoint. In total, 4 participants were identified as outliers above the +2SD. Additionally, 5 participants with dark-colored skin could not be measured with the AGE reader at timepoint one and 4 at timepoint two. In contrast, all participants were able to undergo SAF analysis with the AGE sensor, irrespective of their skin type. Conclusions: Both machines showed excellent intrarater reliability and two-timepoint agreement, but the skin AGE reader might have limited applicability in individuals with dark-colored skin. Future research on AGEs might take our findings into consideration.

## 1. Introduction

The heterogeneous group of advanced glycation end products (AGEs) are formed as a result of non-enzymatic attachment of glucose to proteins such as type I collagen, lipids, or nucleic acids through Maillard’s reaction [1,2,3,4]. Tissues undergo unfavorable structural and functional modifications when AGEs accumulate, and subsequent binding to the receptor for advanced glycation end products (RAGE) occurs [3,5]. The accumulation of AGEs on tissue proteins has been implicated in playing a critical role in various settings such as aging, diabetes, cardiovascular diseases, Alzheimer’s disease, and acute or chronic oxidative stress such as renal failure [6,7,8]. In addition to these conditions, AGEs have also been shown to accumulate in bone and are gaining interest in connective tissue research. Studies suggest that extracellular matrices made out of long-living proteins such as skeletal muscles, are highly vulnerable to AGE modifications, muscle strength, and muscle mass [9,10,11,12]. Additionally, cross-sectional studies have demonstrated a destructive effect of AGEs on bone properties and reduced bone material strength index (BMSi), which are potential measures of bone quality [2,3,5,13,14]. However, the role of AGEs in bone metabolism or osteoporosis is not completely understood [2]. 

Previously, AGE accumulations were assessed by using skin biopsies, which are not suitable for routine clinical use due to their degree of invasiveness [5]. Recently, commercially available, non-invasive, and user-friendly devices measuring AGEs by using SAF or percutaneous fluorescence (fingertip) were validated and referenced against specific AGE levels in skin biopsy specimens or AGEs circulating in the blood (fingertip) [3,7,15]. However, there is no defined gold standard on the optimal measurement tool and on which body part AGEs should be measured with SAF, although measurement using SAF is considered a promising non-invasive technique [7,16,17].

The aim of this study was to investigate the intrarater reliability, two-timepoint agreement, and correlations within and between two widely used and commercially available SAF AGE machines in a healthy adult volunteer population.

## 2. Material and Methods

### 2.1. Ethics Statement

The study protocol was approved by our hospital’s institutional review board (#2014-084). Informed written consent was obtained from all participants, and the study was conducted according to the principles expressed in the Declaration of Helsinki. 

### 2.2. Study Design and Setting

Between February and March 2021, healthy volunteers aged 18 years and older were recruited at a single academic institution and enrolled in this study. Each participant underwent SAF reader analysis by using both the Advanced Glycation Endproduct AGE reader Version 2.3 (DiagnOptics Technologies BV, Groningen, The Netherlands) and the AGE sensor Model: RQ-1101J-SET (Air Water Biodesign Co., Kobe, Japan) which are both approved by regulatory authorities. All AGE measurements on both devices were performed by two researchers in the same manner and in accordance with the manufacturer’s operational guides. No specialized training was required to operate the devices. Measurements were repeated three times for each SAF reader on two separate occasions 14 to 30 days apart. Additionally, at timepoint one, a general survey about the participant’s lifestyle and health status was completed and administered again to identify any changes at timepoint two. Participants with incomplete measurements or health survey data were excluded from the final analysis. 

### 2.3. Technique of Skin Autofluorescence Advanced Glycation End Products Measurement

The AGE reader Version 2.3 (DiagnOptics Technologies BV, Groningen, The Netherlands), hereinafter referred to as “AGE reader”, is a fully automated version of an autofluorescence reader with a built-in spectrometer that uses the volar side of the forearm as the location of measurement [18]. Studies showed that SAF values measured by the AGE reader have moderate and strong correlations with values derived from skin biopsy (r = 0.47 and 0.55 for Nε-(carboxyethyl)lysine and pentosidine, respectively), making this device a noninvasive and easy method for analyzing AGEs [7,18,19]. As the excitation light source, the AGE reader utilizes ultraviolet A (UVA) with a peak wavelength at 370 nm that illuminates 4 cm^2^ of skin. In general, emission light with a wavelength of 420–600 nm and reflected excitation light of 300–420 nm from the skin are measured and the ratio of excitation light to emitted light is calculated and reported in arbitrary numerical units (AU) [2,5,7]. 

The second device used in our study was the AGEs sensor model: RQ-1101J-SET (Air Water Biodesign, Co., Kobe, Japan), hereinafter referred to as “AGE sensor”. In contrast to the AGE reader, the fully automated percutaneous fluorescence AGEs sensor measures the amount of AGEs in the serum from the fingertip of the non-dominant index finger indirectly. The AGEs sensor correlates *Nδ*-(5-hydro-5-methyl-4-imidazolone-2-yl)-ornithine (MG-H1) circulating in the serum of the blood to the unique light emitted from the skin of the fingertip as described by Yamanaka et al. [20] The AGE Sensor analyzes the fluorescence emission spectrum of 440–460 nm from an approximate 0.38 mm diameter area of the fingertip [9].

### 2.4. Intrarater Reliability, Two-Timepoint Agreement and Correlations 

For intrarater reliability and two-timepoint agreement, the continuous AGE scores in AU were used. Intrarater reliability of the AGE scores was performed within a single series of 3 consecutive measurements at timepoint one for each machine as well as for the average of all 3 measurements. Furthermore, interclass correlation coefficients (ICCs) for two-timepoint agreement over time was calculated for each machine between timepoint one and two for the AGEs scores. Afterwards, correlations between the two devices and the two timepoints within one device were analyzed by using the AGE scores and outliers (+2SD) were evaluated. 

### 2.5. Statistical Analysis

For continuous AGEs scores, ICC estimates with 95% confidence intervals for intrarater reliability were calculated based on mean rating (k = 3), absolute agreement, and two-way random effects models. ICC estimates with 95% confidence intervals for two-timepoint agreement were calculated based on mean rating (k = 2), absolute agreement, and two-way random effects models. Linear regression models were used to examine the correlation of the scores between the two devices and correlations between two timepoints within one device. Scatter plots with regression lines were generated to visualize the correlations. Outliers were identified by using 2 standard deviations of the residuals. The interpretation of ICC was done according to Koo and Li [21]. Statistical analysis was performed by using SAS version 9.4 (SAS Institute Inc., Cary, NC, USA). The statistical significance level was set as *p* ≤ 0.05.

## 3. Results

### 3.1. Participant’s Demographics and Characteristics

Demographic characteristics are summarized in Table 1. A total of 42 participants were enrolled in the study, of which two were excluded due to a follow-up measurement that was outside the 30-day upper visit window, resulting in 40 participants included in the final analysis. The second AGE measurement was completed on average 14.7 ± 1.7 days (range 14–21 days) after the initial measurement. The study population consisted of 25 female and 15 male participants, with an average age of 39 ± 12.5 years (range 21–71 years). The predominantly White/Caucasian population (52.5%) had a Fitzpatrick skin type (FPST) II (32.5%) (I lightest to VI darkest) most often and an average BMI of 25.9 ± 5.2 kg/m^2^ [22]. 

The lifestyle and health status characteristics based on the survey are displayed in Table 2. Participants who regularly used skin moisturizer did so on average 7.3 ± 3.7 times a week. Participants who regularly used anti-aging products did so on average 6.8 ± 3.9 times a week, and participants who reported regular physical activity exercised on average 3.6 ± 2.3 days a week. At the follow-up timepoint, 9 participants stated that they changed their general lifestyle: 2 did more exercise, 2 did less exercise, 2 started a healthier lifestyle, 1 reported an unhealthier lifestyle, 1 went to a tanning salon, and 1 reported serious health issues that were not further specified. 

At timepoint one, the AGE reader was unable to measure 5 participants with dark skin: 3 had a FPST V and 2 had skin type VI. There were no measurement issues in the same participants at this timepoint by using the AGE sensor. At timepoint two, 4 of these participants had the same issue using the AGE reader and just one (skin type V) could be measured. For the AGE sensor, all measurements could be completed at timepoint two.

### 3.2. Intrarater Reliability and Two-Timepoint Agreement

For the AGE reader scoring, intrarater reliability was excellent at timepoint one (ICC = 0.998, 95%CI 0.996–0.999) and timepoint two (ICC = 0.996, 95% CI 0.993–0.998). The intrarater reliability for the AGE sensor scoring was also excellent when comparing the three consecutive measurements at timepoint one (ICC = 0.942, 95% CI 0.904–0.967) and timepoint two (ICC = 0.923, 95%CI 0.866–0.957) (Table 3(1)). 

The two-timepoint agreement for average AGE score was excellent for both readers, with an ICC estimate of 0.965 (95%CI 0.931–0.982) for the AGE reader and an ICC estimate of 0.912 (95%CI 0.834–0.953) for the AGE sensor (Table 3(2)). 

### 3.3. Correlation Analyses

**Figure 1:**
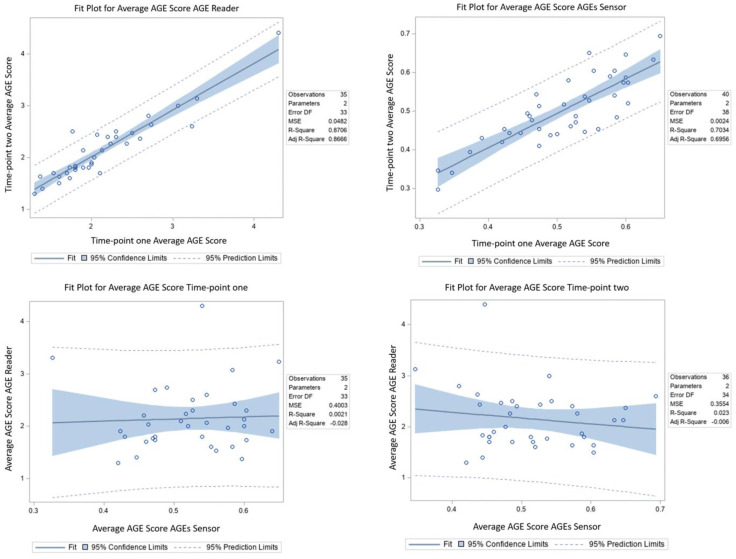
Correlation plots within (**top**) and between (**bottom**) AGE reader and AGE sensor.

When looking at the correlation between the average score at timepoint one and timepoint two using the AGE reader, strong correlations (*p* < 0.001) were observed in two participants considered outliers with +2SD (Figure 1). One participant was a 25-year-old White/Caucasian (FPST III) female with a BMI of 22.2 kg/m^2^ and no preexisting medical conditions. She regularly used skin moisturizer 5×/week, anti-aging products 1×/week and did not do any regular physical activity. However, she stated that during the 14-day period between two measurements, she went to a tanning salon for a spray tan. The other outlier was a 71-year-old female (FPST II) with a BMI of 25.6 kg/m^2^ and no preexisting medical conditions. She used skin moisturizer daily and did not use anti-aging products or perform regular physical activity. However, she specified that between both timepoints, she consumed more alcohol than usual because of birthday celebrations. 

Regarding the correlation between the average score at timepoint one and timepoint two using the AGE sensor, a strong correlation (*p* < 0.001) was also present (Figure 1), and one participant was considered an outlier above the +2SD. This 50-year-old Asian-American (FPST IV) male with a BMI of 31.6 kg/m^2^ had no preexisting medical conditions, used skin moisturizers twice a week, and did regular physical activity twice a week. He did not state any changes in lifestyle between both measurements. 

When analyzing the correlation between the AGE reader and AGE sensor for each timepoint, there were no statistically significant correlations found for either timepoint (timepoint one *p* = 0.795; timepoint two *p* = 0.377) (Figure 1). In total, there was one outlier above the +2SD at both timepoints. This 39-year-old African-American female (FPST V) had a BMI of 39.5 kg/m^2^ and no preexisting medical conditions. She regularly used skin moisturizer 2×/day, did not use anti-aging products, and had regular physical activity (walking) 10×/week. According to the follow-up questionnaire, she drank more water between both measurements..

## 4. Discussion

This study is the first to directly compare the intrarater reliability and two-timepoint agreement among two commercially available SAF AGE readers. Furthermore, we analyzed the correlation within and between these two machines. Intrarater agreement at one timepoint and test–retest reliability over two timepoints were excellent or good for both devices. Correlations within each machine were strong over time, but when comparing both machines at different timepoints, no correlations were found.

AGE SAF readers are used to screen patients for a variety of conditions. Multiple studies have demonstrated possible areas for their clinical application, ranging from diabetes and chronic diabetic complications, cardiovascular risk analysis, renal diseases, and musculoskeletal related diseases such as osteoporosis [3,5,9,23,24]. Particularly for diabetes and diabetes-related micro- and macro-vascular complications, there is strong evidence that SAF is directly correlated with the severity of these conditions and therefore represents an additional useful and non-invasive screening tool [3].

When performing risk factors analysis for diseases by using SAF devices, the patient’s age must be considered. Multiple studies have suggested that AGEs increase with age when measured in different body parts [25,26]. Koetsier et al. [18] compiled reference values of SAF for each decade of life. Their results showed a constant degree of variation, and SAF values can be described as a linear function of age up to 70 years of age. 

Additionally, when using AGE SAF devices for screening, it is important to perform the measurements at the same body regions. Fernando et al. [19] analyzed the within- and between-body-site agreement when performing SAF AGE measurements. Their results showed a substantial to almost perfect agreement by measuring at the same body site and poor agreement when measuring at different body sites. The authors argued that in addition to other factors such as skin translucency, there might be variations in glycation at different body regions leading to the discrepancies. Other studies showed that there were larger differences when measuring at different body sites compared to measuring patient populations with or without diabetes, which highlights the need for a consistent measurement protocol based on body site [27,28]. Furthermore, previous studies have shown excellent repeatability of SAF measurements when done on the same machine and at the same body part with low coefficients of variation (up to 10.9%) for measurements repeated up to 12 weeks [7,29].

The results of our study are in agreement with previous AGE reader studies. When measuring at the same body part using the same machine, both fully automated readers showed excellent intrarater (series of 3 measurements) and two-timepoint agreement (repeated measurement after 2–4 weeks), with results using the AGE reader showing only minimal deviations from the previous measurement compared to the AGE sensor. This is most likely due to the different AGEs each device measures. The AGE reader analyzes the accumulation of AGEs in the skin of the forearm, which has been proposed as a measure of metabolic history and can therefore be less prone to changes over a short period of time [30]. On the other hand, the AGE sensor uses an indirect way to analyze AGEs in the blood and therefore might be more sensitive to changes over a short period of time, potentially explaining the difference in test–retest reliability found in our study. The fact that both devices analyze different AGEs is supported by the results of our correlation assessment. Because the AGE reader and AGE sensor measure different AGEs, no correlations were found at either of the two timepoints between both devices.

Another interesting finding of our study was related to skin color. Up until now, the majority of AGE reader studies and device validation were based on Caucasian or Asian populations with an FPST skin type I–IV. SAF analysis can be impaired in patients with darker skin and a skin reflectance percentage (SR%) of less than 10% due to the photoprotective role of melanin against UV radiation in the dermis [3,31]. However, it is essential for AGE devices to have the capability to perform SAF analysis in populations with dark skin because it represents the most prevalent skin type in the world in increasing number and constitutes the population with the highest incidence of diabetes [32]. In our healthy volunteer group, a total of 7 participants had a FPST V or VI of which 5 (2 type VI and 3 type V) had technical issues when using the AGE reader at timepoint one and 4 (2 type VI and 2 type V) at timepoint two. In contrast, all participants were able to undergo SAF analysis with the AGE sensor, irrespective of their skin type. This finding regarding natural skin type should be taken into consideration for future studies involving subjects with dark skin. 

Another important finding was observed when looking at the correlations within and between the AGE machines. For measurements between timepoint one and two within the AGE reader, there were two outliers above the +2SD. The first outlier underwent a spray tan between the two measurements. This discrepancy in measurement, however, was only present in the AGE reader and not in the AGE sensor. This significant change resulting from artificial skin-color changes should be taken into consideration when interpreting AGE results. In the same context, changes in skin color that might occur due to physiological or artificial tanning should be kept in mind when comparing the results of two measurements. The second outlier in the abovementioned setting was 71 years old. This participant was above the reference values of SAF that Koetsier et al. [18] suggested, which might explain why his AGE reader score was above +2SD. Similar to the first outlier, this participant was not considered an outlier in the AGE sensor. The two other outliers (one for measurement between both timepoints and within the AGE sensor and one between the AGE reader and AGE sensor for both timepoints) did not show any specific distinguishing factors and therefore should be interpreted with caution. It cannot be ruled out that there are unmeasured factors that potentially favor scores above the +2SD. 

There are several limitations to our study. First, our cohort represents a rather heterogeneous group with a relatively small sample size. Second, the study lacked a comparison group of non-healthy participants. The healthy volunteers had no or very few chronic health conditions, which made assessments between healthy participants and participants with chronic conditions not possible. Third, participants were not instructed to change any of their habits during the study period, but the results of the first three measurements was not blinded to the patients and might have affected their lifestyle.

## 5. Conclusions

To the author’s knowledge, this is the first study to analyze the intrarater reliability and two-timepoint agreement as well as correlations of two commercially available and widely used AGE readers. The reliability for both readers was excellent when analyzing the AGE scores and there were strong correlations within each device over time, but not when comparing scores between devices at either timepoint. Furthermore, there seems to be limited applicability of the forearm AGE reader in individuals with dark skin (Fitzpatrick skin type V and VI) and slight artificial changes of skin color need to be considered when interpreting repeat AGE measurements. 

## Figures and Tables

**Table 1:** Demographic characteristics (n = 40).

Variables	
Gender, n (%)	
Male	15 (37.5)
Female	25 (62.5)
Race, n (%)	
White/Caucasian	21 (52.5)
Others	8 (20.0)
Black/African American	6 (15.0)
Asian	5 (12.5)
Mean Body Mass Index (BMI) (kg/m^2^), (SD)	25.9 (5.2)
Fitzpatrick Skin Type, n (%)	
I	4 (10.0)
II	13 (32.5)
III	9 (22.5)
IV	7 (17.5)
V	5 (12.5)
VI	2 (5.0)

**Table 2:** Lifestyle and health status of study participants (n = 40). * This question was only used at timepoint two.

Variables, n (%)	
Osteoporosis	0 (0.0)
Diabetes	1 (2.5)
Chronic Heart Disease	0 (0.0)
Chronic Lung Disease	1 (2.5)
Kidney Disease	0 (0.0)
Liver Disease	0 (0.0)
Mental Health Problems	0 (0.0)
Smoking	
Never	36 (90.0)
Former	3 (7.5)
Current	1 (2.5)
Alcohol consumption	
Never	3 (7.5)
Social drinker	29 (72.5)
More	6 (15.0)
N/A	2 (5.0)
Regular usage of skin moisturizer	33 (82.5)
Regular usage of anti-aging products	13 (32.5)
Physical activity within the last 48 h	20 (50.0)
Regular physical activity	25 (62.5)
Lifestyle changes since the first measurement *	9 (22.5)

**Table 3:** (**1**). ICC for intrarater reliability.(**2**). ICC for test–retest reliability.

(**1**). ICC for intrarater reliability		
**ICC for intrarater reliability**		
**Value**	**ICC**	**95% CI**
AGE reader		
Timepoint 1	0.998	(0.996, 0.999)
Timepoint 2	0.996	(0.993, 0.998)
AGE sensor		
Timepoint 1	0.942	(0.904, 0.967)
Timepoint 2	0.923	(0.866, 0.957)
(**2**). ICC for test–retest reliability		
**ICC for two-timepoint agreement**		
**Value**	**ICC**	**95% CI**
AGE reader		
1. Measurement	0.958	(0.919, 0.979)
2. Measurement	0.969	(0.939, 0.984)
3. Measurement	0.965	(0.923, 0.980)
Average score	0.965	(0.931, 0.982)
AGE sensor		
1. Measurement	0.848	(0.711, 0.919)
2. Measurement	0.814	(0.647, 0.901)
3. Measurement	0.861	(0.723, 0.928)
Average score	0.912	(0.834, 0.953)

## Data Availability

The datasets generated during and/or analyzed during the current study are available from the corresponding author on reasonable request.
